# Time to first cigarette after waking and incident heart failure: a dose-response analysis from the UK biobank

**DOI:** 10.1093/eschf/xvag049

**Published:** 2026-02-18

**Authors:** Jiahua Wen, Zhaohan Yan, Yue Cao, Huisong Ma, Yunying Zeng, Ling Zhao, Jianzhao Su, Yunzhao Hu, Yuli Huang

**Affiliations:** Department of Cardiology, The Eighth Affiliated Hospital, Southern Medical University (The First People’s Hospital of Shunde) Foshan, No. 1 Jiazi Road, Lunjiao, Shunde District, Foshan City, Guangdong Province 528308, China; Medical Research Center, The Eighth Affiliated Hospital, Southern Medical University (The First People's Hospital of Shunde), No. 1 Jiazi Road, Lunjiao, Shunde District, Foshan City, Guangdong 528308, China; Department of Cardiology, The Eighth Affiliated Hospital, Southern Medical University (The First People’s Hospital of Shunde) Foshan, No. 1 Jiazi Road, Lunjiao, Shunde District, Foshan City, Guangdong Province 528308, China; Medical Research Center, The Eighth Affiliated Hospital, Southern Medical University (The First People's Hospital of Shunde), No. 1 Jiazi Road, Lunjiao, Shunde District, Foshan City, Guangdong 528308, China; Department of Cardiology, The Eighth Affiliated Hospital, Southern Medical University (The First People’s Hospital of Shunde) Foshan, No. 1 Jiazi Road, Lunjiao, Shunde District, Foshan City, Guangdong Province 528308, China; Medical Research Center, The Eighth Affiliated Hospital, Southern Medical University (The First People's Hospital of Shunde), No. 1 Jiazi Road, Lunjiao, Shunde District, Foshan City, Guangdong 528308, China; Department of Cardiology, The Eighth Affiliated Hospital, Southern Medical University (The First People’s Hospital of Shunde) Foshan, No. 1 Jiazi Road, Lunjiao, Shunde District, Foshan City, Guangdong Province 528308, China; Medical Research Center, The Eighth Affiliated Hospital, Southern Medical University (The First People's Hospital of Shunde), No. 1 Jiazi Road, Lunjiao, Shunde District, Foshan City, Guangdong 528308, China; Department of Cardiology, The Eighth Affiliated Hospital, Southern Medical University (The First People’s Hospital of Shunde) Foshan, No. 1 Jiazi Road, Lunjiao, Shunde District, Foshan City, Guangdong Province 528308, China; Medical Research Center, The Eighth Affiliated Hospital, Southern Medical University (The First People's Hospital of Shunde), No. 1 Jiazi Road, Lunjiao, Shunde District, Foshan City, Guangdong 528308, China; Department of Cardiology, The Eighth Affiliated Hospital, Southern Medical University (The First People’s Hospital of Shunde) Foshan, No. 1 Jiazi Road, Lunjiao, Shunde District, Foshan City, Guangdong Province 528308, China; Medical Research Center, The Eighth Affiliated Hospital, Southern Medical University (The First People's Hospital of Shunde), No. 1 Jiazi Road, Lunjiao, Shunde District, Foshan City, Guangdong 528308, China; Department of Cardiology, The Eighth Affiliated Hospital, Southern Medical University (The First People’s Hospital of Shunde) Foshan, No. 1 Jiazi Road, Lunjiao, Shunde District, Foshan City, Guangdong Province 528308, China; Medical Research Center, The Eighth Affiliated Hospital, Southern Medical University (The First People's Hospital of Shunde), No. 1 Jiazi Road, Lunjiao, Shunde District, Foshan City, Guangdong 528308, China; Department of Cardiology, The Eighth Affiliated Hospital, Southern Medical University (The First People’s Hospital of Shunde) Foshan, No. 1 Jiazi Road, Lunjiao, Shunde District, Foshan City, Guangdong Province 528308, China; Medical Research Center, The Eighth Affiliated Hospital, Southern Medical University (The First People's Hospital of Shunde), No. 1 Jiazi Road, Lunjiao, Shunde District, Foshan City, Guangdong 528308, China; Department of Cardiology, The Eighth Affiliated Hospital, Southern Medical University (The First People’s Hospital of Shunde) Foshan, No. 1 Jiazi Road, Lunjiao, Shunde District, Foshan City, Guangdong Province 528308, China; Medical Research Center, The Eighth Affiliated Hospital, Southern Medical University (The First People's Hospital of Shunde), No. 1 Jiazi Road, Lunjiao, Shunde District, Foshan City, Guangdong 528308, China; Guangdong Provincial Key Laboratory of Cardiac Function and Microcirculation Research, Department of Cardiology, Southern Medical University, 1023 Shatai South Road, Baiyun District, Guangzhou, Guangdong Province 510515, China; The George Institute for Global Health, Faculty of Medicine, University of New South Wales, Level 5, 1 King Street, Newtown, Sydney, NSW 2042, Australia

**Keywords:** Heart failure, Smoking behaviour, Time to first cigarette, Prospective cohort, Nicotine dependence

## Abstract

**Background and Aims:**

We aimed to investigate the association between time to first cigarette after waking (TTFC) and incident heart failure (HF) in a large prospective cohort.

**Methods:**

This study included 229 391 participants from the UK Biobank. Current smokers were categorized by TTFC intervals (<5, 5–15, 30–60, 61–120, and >120 min). Multivariable Cox proportional hazards models were employed to assess the relationship between TTFC and HF. Joint analyses evaluated the combined associations of TTFC with daily cigarette amount and smoking duration. Subgroup and interaction analyses were conducted by age, sex, BMI, education, and alcohol consumption.

**Results:**

During a median follow-up of 15.5 years, 6912 (3.01%) incident HF cases occurred among 229 391 participants. The cohort included 203 653 (88.77%) non-smokers (age 55.9 ± 8.1 years, 41.4% female, 91.5% White ethnicity, 2.7% incident HF cases) and 25 738 (11.23%) current smokers (age 54.5 ± 8.1 years, 49.0% female, 89.1% White ethnicity, 5.7% incident HF cases). A significant dose-dependent relationship was observed between shorter TTFC and HF risk (*P* for trend <.001). Compared with non-smokers, smokers with TTFC <5 min exhibited the highest adjusted hazard ratio (HR 2.22, 95% CI 1.58–3.10; *P* < .001), corresponding to an absolute risk difference of 4.03%. Joint analyses showed that among individuals smoking ≤median cigarettes/day, HF risk increased from HR 1.61 (95% CI: 1.35–1.92; *P* < .001) to 2.25 (1.86–2.72; *P* < .001) across decreasing TTFC categories; among those smoking >median cigarettes/day, the corresponding HRs ranged from 1.95 (1.41–2.69; *P* < .001) to 2.17 (1.74–2.69; *P* < .001). Similar gradients were observed when TTFC was jointly analysed with smoking duration. Subgroup analyses indicated stronger associations in participants aged <60 years (HR 1.98, 95% CI 1.56–2.51; *P* < .001 for TTFC <15 min) than in those ≥60 years (HR 1.55, 95% CI 1.25–1.91; *P* < .001), both compared with non-smokers (*P* for interaction <.001).

**Conclusions:**

Shorter TTFC is independently and dose-dependently associated with a higher risk of incident HF, even after accounting for smoking burden and comorbidities. TTFC assessment may improve HF risk stratification, particularly in younger individuals.

## Graphical Abstract

**Graphical Abstract:**
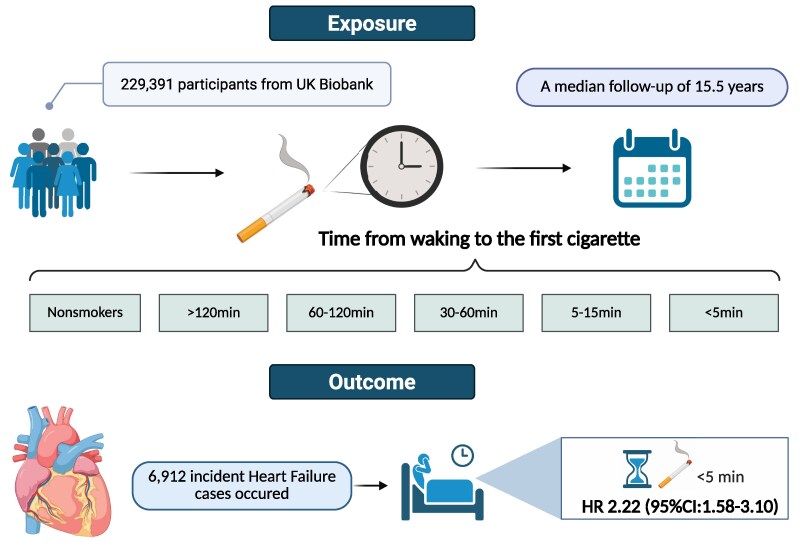

## Introduction

Heart failure (HF), a complex clinical syndrome representing the end-stage progression of cardiovascular disease (CVD), is characterized by high global incidence and mortality, imposing a substantial socioeconomic burden.^1^  ^,2^ While traditional risk factors like hypertension and coronary artery disease are well established,^3^ modifiable behavioural factors such as smoking warrant further investigation regarding their temporal characteristics.

Cigarette smoking is a well-established risk factor for CVD, contributing to HF development through chronic inflammation, oxidative stress, endothelial dysfunction, autonomic imbalance, and adverse myocardial remodelling.^4^ Recent evidence suggests this relationship extends beyond cumulative exposure, exhibiting distinct chronobiological patterns.^5^ The time to first cigarette after waking (TTFC) has emerged as an important behavioural biomarker, where shorter intervals reflect more severe nicotine dependence involving up-regulated nicotinic acetylcholine receptor signalling and altered dopamine reward pathways.^6,7^ Mechanistically, this accelerated morning nicotine intake may exacerbate cardiovascular stress via acute sympathetic activation, endothelial dysfunction from oxidative stress, and circadian disruption of cardiac metabolic rhythms.^8,9^

Recent trends indicate that smokers today are increasingly likely to smoke their first cigarette earlier in the morning than smokers a decade ago.^6^ Moreover, shorter TTFC has been associated with atrial fibrillation,^5^ a higher prevalence of hypertension,^10^ and lower high-density lipoprotein (HDL) levels,^11^ after accounting for daily cigarette consumption. As atrial fibrillation, elevated blood pressure, and reduced HDL are well-established contributors to cardiovascular risk, including incident HF,^12–14^ we hypothesized that shorter TTFC may be independently associated with an increased risk of incident HF.

Therefore, we conducted a prospective cohort analysis to investigate the association between TTFC and incident HF risk. Our findings may help advance understanding of smoking-related HF pathophysiology and provide evidence supporting targeted cessation interventions that focus on early morning smoking behaviour.

## Methods

### Study design and participants

**Figure 1:**
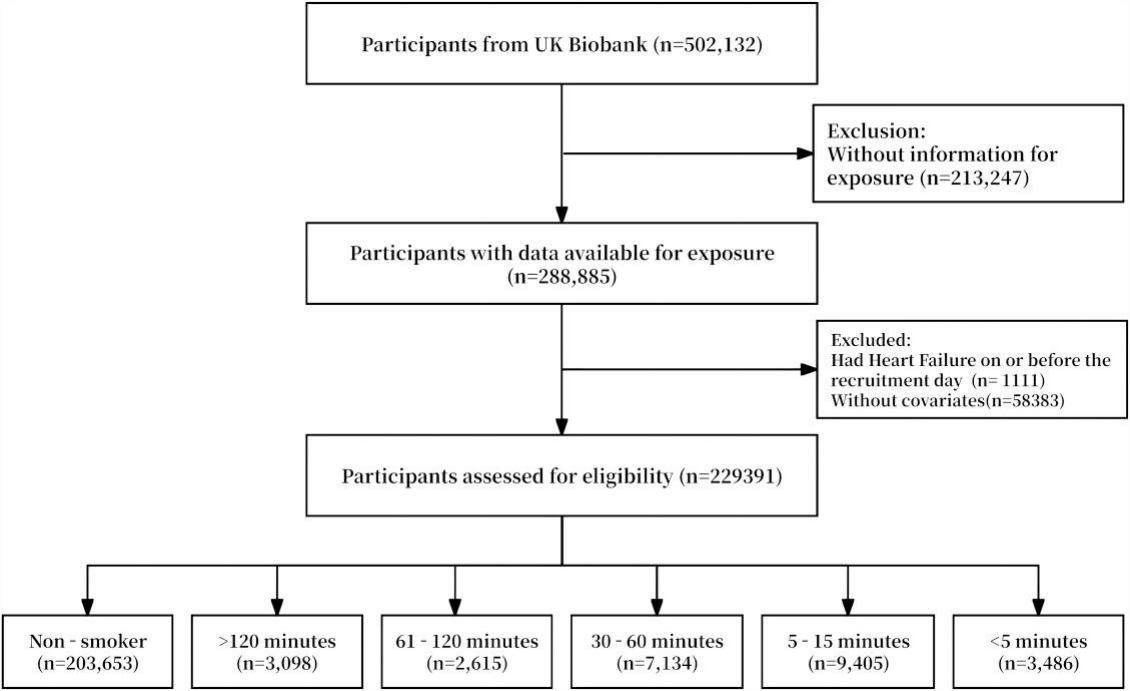
Participant selection flow chart. From the initial UK Biobank cohort (*n* = 502 132), we excluded participants with missing exposure data (*n* = 213 247), prevalent heart failure (*n* = 1111), and incomplete covariate information (*n* = 58 383), yielding a final analytical sample of 229 391 individuals

The UK Biobank is a large-scale population-based cohort study that recruited over 500 000 participants aged 40–69 years between 2006 and 2010. Participants were enrolled from 22 assessment centres across England, Wales, and Scotland. It was approved by the Community Health Index Advisory Group in Scotland, the North West Multi-Centre Research Ethics Committee, and the National Information Governance Board for Health and Social Care in England and Wales. All participants completed informed consent at baseline. From the initial 502 132 participants, we excluded 213 247 with missing exposure data, 1111 with prevalent HF, and 58 383 with incomplete covariate information, yielding a final analytical cohort of 229 391 participants (*Figure 1*).

### Exposure and covariates assessment

Smoking behaviour was assessed via baseline touchscreen-based questionnaire, with TTFC measured by asking ‘How soon after waking do you typically smoke your first cigarette?’ using five pre-defined intervals (<5, 5–15, 30–60, 61–120, or >120 min), creating six mutually exclusive categories including non-smokers as reference.^5^

### Covariates

Detailed information can be obtained from the UK Biobank website (www.ukbiobank.ac.uk).

Age, sex, and daily cigarette consumption were self-reported. Ethnicity was categorized as white or other ethnic groups. The Townsend deprivation index (TDI) is a composite measure that includes factors such as unemployment, non-car ownership, non-home ownership, and household overcrowding to assess social deprivation, with higher scores indicating greater deprivation.^15^ Educational attainment was classified as having a college or university degree or other educational levels. Alcohol consumption status was categorized into current, past, or never. We calculated the body mass index (BMI) by dividing weight in kilograms by the square of height in metres (kg/m²). The waist-to-hip ratio (WHR) was obtained by dividing the waist circumference by the hip circumference. Self-reported sleep duration was categorized as healthy (7–8 h/day), unhealthy (≤6 or ≥9 h/day). Systolic blood pressure (SBP) and diastolic blood pressure (DBP) were the average of two measurements. Participant characteristics also included glycated haemoglobin (HbA1c), blood glucose (Glu), white blood cells (WBC), neutrophils (NEU), lymphocytes (LYM), C-reactive protein (CRP), low-density lipoprotein cholesterol (LDL-C), triglycerides (TG), and estimated glomerular filtration rate (eGFR). The CKD Epidemiology Collaboration (CKD-EPI) equation was used to estimate the eGFR.^16^ Diabetes was defined based on the onset time of diabetes (before or equal to the date of attending the assessment centre) or self-reported history of doctor-diagnosed diabetes. Hypertension was defined as the use of antihypertensive medications or doctor-reported diagnosis of hypertension. This definition was chosen to capture clinically recognized and treated hypertension, as relying solely on baseline blood pressure measurements may miss cases of masked hypertension or intermittently elevated blood pressure. The use of glucose-lowering, lipid-lowering, and antihypertensive medications, as well as baseline diseases such as heart disease, angina, and stroke, were confirmed by self-report.

### Outcome ascertainment

The primary study outcome was incident HF, determined by ICD-10 diagnosis codes I11.0, I13.0, I13.2, and I50 from inpatient records.^15^ These records included admission and diagnosis data from the Hospital Episode Statistics for England, Scottish Morbidity Record data for Scotland, and the Patient Episode Database for Wales. The follow-up duration was calculated from the baseline date to the incidence of HF, death, or the censoring date (1 September 2024), whichever occurred first.

### Statistical analysis

Continuous variables were expressed as mean (SD), and categorical variables were presented as counts and percentages. Normality was assessed using the Shapiro–Wilk test. For the comparison of continuous variables between groups, the Kruskal–Wallis test (for non-normal distribution) or one-way analysis of variance (for normal distribution) was selected according to the characteristics of data distribution. The χ^2^ test was used for the comparison of categorical variables. Missing data were handled using a complete-case analysis.

The association between TTFC and HF was assessed by three Cox proportional hazards regression models: Model 1 was not adjusted for confounding factors; Model 2 was adjusted for demographic and clinical variables (age, sex, TDI, ethnicity, educational attainment, BMI, eGFR, WHR, alcohol consumption status, SBP, DBP, hypertension, diabetes, WBC, NEU, LYM, CRP, TG, HbA1c, fasting blood glucose, LDL-C, sleep status, CVD history, and medication history); Model 3 was further adjusted for daily cigarette consumption and smoking duration based on Model 2. The proportional hazards assumption was evaluated using Schoenfeld residuals. The Kaplan–Meier method was used to generate survival curves, and the log-rank test was used to compare the differences in the probability of HF occurrence among different smoking time groups. In addition, we performed a series of subgroup analyses by age (<60 years or ≥60 years), sex (female or male), ethnic background (other or white), education (having a college or university degree or other educational levels), BMI (<25, 25–30, or ≥30 kg/m²), and alcohol consumption status (current, past, or never). We used the same Cox model and added interaction terms. These subgroup analyses were exploratory in nature and were not adjusted for multiple comparisons.

In addition, we performed joint analysis of smoking timing with daily cigarette amount and smoking duration on the risk of incident HF. Moreover, we used an XGBoost model and SHAP values to rank feature importance for incident HF. The XGBoost model was used as a complementary, exploratory analysis, and not for causal inference. To assess the stability of the results, we performed five sensitivity analyses. First, we excluded participants who had an onset of HF within 2 years. Second, we excluded participants with heart disease or angina at baseline. Third, we excluded participants with stroke at baseline. Fourth, we replaced daily cigarette consumption and smoking duration with pack-years to evaluate whether the TTFC-HF association remained consistent when using an alternative composite measure of cumulative smoking exposure. Fifth, we used a Fine—Gray competing risk model, with all-cause mortality and non-cardiovascular mortality as the competing events, to further assess the association between TTFC and incident HF. All statistical analyses were performed using R 4.4.1. A two-sided *P*-value <.05 was considered statistically significant.

## Results

### Baseline characteristics

The study population comprised 229 391 participants (203 653 non-smokers and 25 738 current smokers stratified by TTFC). Smokers with shorter TTFC intervals displayed a distinct risk profile, being younger, with higher female representation (54.8% in the <5-min group), and a greater proportion of other levels of education, yet demonstrating significantly worse cardiovascular risk markers including elevated daily cigarette consumption, longer smoking duration, higher inflammatory markers, and greater prevalence of hypertension, diabetes, and established CVD, alongside reduced optimal sleep duration compared with non-smokers (*Table 1*).

**Table 1:** Baseline characteristics of study participants stratified by smoking status and time to first cigarette (TTFC) intervals

Variable	Non-smoker (*n* = 203 653)	Time from waking to first cigarette (min)					*P*-value
		>120 (*n* = 3098)	61–120 (*n* = 2615)	30–60 (*n* = 7134)	5–15 (*n* = 9405)	<5 (*n* = 3486)	
Demographic characteristics							
Age, years	55.86 (8.14)	53.25 (8.17)	54.34 (8.28)	55.19 (8.13)	54.72 (7.91)	53.35 (7.60)	<.001
Townsend deprivation index	−1.70 (2.86)	−0.24 (3.36)	−0.43 (3.38)	−0.09 (3.38)	0.49 (3.48)	1.47 (3.57)	<.001
Sex, %							<.001
Male	119 305 (58.58)	1783 (57.55)	1312 (50.17)	3658 (51.28)	4810 (51.14)	1577 (45.24)	
Female	84 348 (41.42)	1315 (42.45)	1303 (49.83)	3476 (48.72)	4595 (48.86)	1909 (54.76)	
Race, %							
White ethnicity	186 326 (91.49)	2668 (86.12)	2315 (88.53)	6414 (89.91)	8459 (89.94)	3088 (88.58)	<.001
Other ethnicity	17 327 (8.51)	430 (13.88)	300 (11.47)	720 (10.09)	946 (10.06)	398 (11.42)	
Education, %							
College university degree	75 068 (36.86)	1015 (32.76)	642 (24.55)	1411 (19.78)	1435 (15.26)	468 (13.43)	<.001
Other level of education	128 585 (63.14)	2083 (67.24)	1973 (75.45)	5723 (80.22)	7970 (84.74)	3018 (86.57)	
Lifestyle factors							
Drinking status, %							
Never	12 211 (6.00)	56 (1.81)	66 (2.52)	206 (2.89)	283 (3.01)	113 (3.24)	<.001
Previous	5717 (2.81)	82 (2.65)	111 (4.24)	376 (5.27)	661 (7.03)	358 (10.27)	
Current	185 725 (91.20)	2960 (95.55)	2438 (93.23)	6552 (91.84)	8461 (89.96)	3015 (86.49)	
Daily cigarette smoked	0.00 (0.00)	6.41 (4.06)	10.69 (5.11)	13.76 (6.08)	17.80 (7.14)	22.71 (10.27)	<.001
Smoking duration, years	0.00 (0.00)	33.85 (10.60)	35.72 (10.13)	36.85 (9.80)	37.42 (9.25)	37.32 (8.77)	<.001
Healthy sleep, %	142 075 (69.76)	2025 (65.36)	1653 (63.21)	4452 (62.41)	5498 (58.46)	1718 (49.28)	<.001
Anthropometric measurements							
BMI, kg/m^2^	27.08 (4.69)	26.44 (4.41)	26.88 (4.70)	26.95 (4.80)	26.75 (4.88)	26.84 (5.18)	<.001
WHR	0.86 (0.09)	0.87 (0.08)	0.88 (0.09)	0.88 (0.09)	0.89 (0.09)	0.90 (0.09)	<.001
SBP	137.30(18.46)	132.24(18.20)	135.14(18.41)	135.80(18.42)	135.65(18.45)	134.78(18.32)	<.001
DBP	82.16 (10.07)	80.68 (10.50)	81.77 (10.12)	81.51 (10.16)	81.53 (10.24)	81.61 (10.24)	<.001
Laboratory biomarkers							
WBC	6.67 (1.90)	7.49 (1.92)	8.07 (2.00)	8.38 (2.08)	8.74 (2.19)	8.96 (2.30)	<.001
NEU	4.09 (1.32)	4.57 (1.56)	5.03 (1.62)	5.26 (1.71)	5.52 (1.79)	5.66 (1.83)	<.001
LYM	1.91 (1.07)	2.15 (0.71)	2.28 (0.72)	2.34 (0.78)	2.42 (0.84)	2.47 (0.97)	<.001
CRP	2.35 (4.03)	2.56 (4.21)	3.05 (4.83)	3.43 (4.88)	3.63 (5.04)	4.16 (5.71)	<.001
TG	1.67 (0.97)	1.86 (1.19)	1.88 (1.14)	1.95 (1.16)	1.97 (1.17)	2.07 (1.26)	<.001
LDL-C	3.58 (0.85)	3.53 (0.85)	3.62 (0.89)	3.63 (0.90)	3.63 (0.92)	3.59 (0.93)	<.001
Glu	5.09 (1.15)	5.00 (1.11)	5.06 (1.24)	5.03 (1.16)	5.05 (1.28)	5.10 (1.36)	<.001
HbA1c	35.62 (6.22)	35.83 (5.53)	36.98 (6.57)	37.41 (6.43)	37.86 (8.57)	38.10 (7.55)	<.001
eGFR	90.89 (13.13)	95.56 (12.80)	94.32 (13.18)	93.83 (13.27)	94.76 (13.18)	96.76 (13.16)	<.001
Medical history and medication							
Diabetes, %	9726 (4.78)	127.00 (4.10)	142 (5.43)	425 (5.96)	630 (6.70)	280 (8.03)	<.001
Hypertension, %	52 329 (25.70)	600 (19.37)	625 (23.90)	1837 (25.75)	2525 (26.85)	1032 (29.60)	<.001
Angina,%	4051 (1.99)	49 (1.58)	70 (2.68)	239 (3.35)	385 (4.09)	192 (5.51)	<.001
Stroke, %	2139 (1.05)	35 (1.13)	44 (1.68)	167 (2.34)	219 (2.33)	119 (3.41)	<.001
Heart attack, %	2405 (1.18)	33 (1.07)	69 (2.640)	200 (2.80)	290 (3.08)	147 (4.22)	<.001
Using lipid-lowering medications, %	26 808 (13.16)	339 (10.94)	356 (13.61)	1211 (16.98)	1723 (18.32)	707 (20.28)	<.001
Using insulin, %	1805 (0.89)	23 (0.74)	31 (1.19)	87 (1.22)	114 (1.21)	52 (1.49)	<.001
Using blood pressure medication,%	17 092 (8.39)	178 (5.75)	213 (8.15)	692 (9.70)	929 (9.88)	410 (11.76)	<.001
Follow-up information							
Incident HF cases, %	5453 (2.68)	97 (3.13)	124 (4.74)	415 (5.82)	589 (6.26)	234 (6.71)	<.001
Person-years	3 056 832	46 098	37 761	101 588	132 234	47 967	

HF, heart failure; BMI, body mass index; WHR, waist—hip ratio; SBP, systolic blood pressure; DBP, diastolic blood pressure; WBC, white blood cell count; NEU, neutrophil count; LYM, lymphocyte count; CRP, C-reactive protein; TG, triglyceride; LDL-C, low-density lipoprotein cholesterol; Glu, glucose; HbA1c, glycated haemoglobin; eGFR, estimated glomerular filtration rate.

Values represent means (standard deviations) for continuous variables and counts (percentages) for categorical variables. *P*-values derive from ANOVA or χ² tests comparing across groups.

### Association between smoking timing and risk of incident heart failure

**Figure 2:**
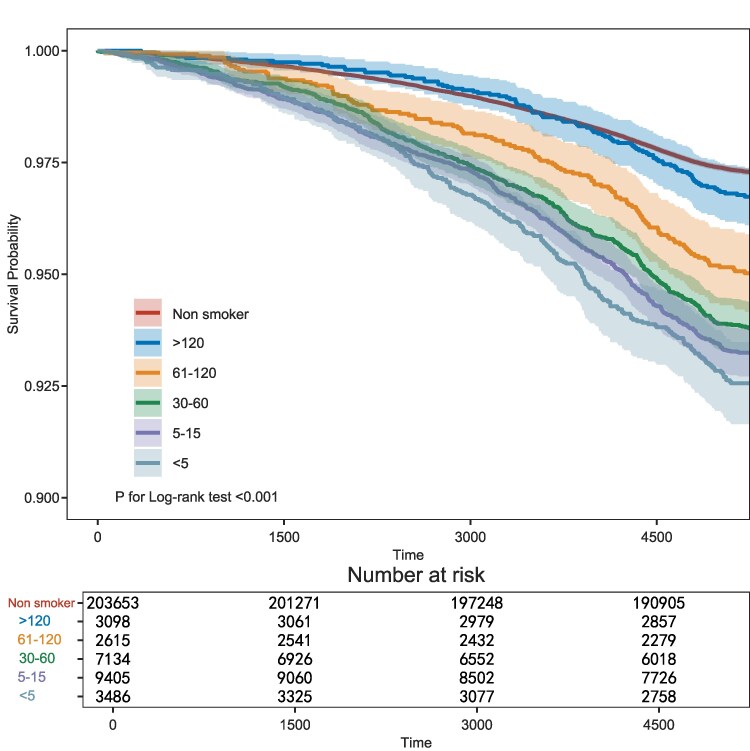
Kaplan–Meier curve for cumulative incidence of heart failure by time to first cigarette (TTFC) group. Log-rank test confirmed significant differences between all TTFC groups (*P* < .001)

During a median follow-up duration of 15.5 years, 6912 incident HF cases occurred. Kaplan–Meier curves demonstrated accelerated survival probability declines with shorter TTFC, particularly for the <5-min group, which diverged markedly from other groups by 4000 days (*Figure 2*). Multivariable Cox proportional hazards models revealed a graded, dose-dependent association between shorter TTFC and increased HF incidence (*P*-trend <.001; *Table 2*). The <5-min TTFC group exhibited the highest risk across all models: yielding an unadjusted hazard ratio (HR) of 2.79 (95% CI: 2.45–3.19) in Model 1, and maintaining a substantially elevated risk (HR = 2.46, 95% CI: 2.12–2.84) in Model 2, after adjustment for 28 covariates encompassing demographic characteristics, metabolic parameters, comorbidities, and medication use. Critically, after further adjustment for daily cigarette consumption and smoking duration in Model 3, the <5-min TTFC group continued to demonstrate a 2.22-fold increased risk (95% CI: 1.58–3.10). A consistent monotonic risk gradient was observed throughout the TTFC categories, as evidenced by the stepwise increase in HRs from the >120-min reference group (Model 3 HR = 1.45) to the <5-min group (*Table 2*). Compared with non-smokers, individuals who smoked within 5 min of waking had an absolute risk difference of 4.03% for incident HF. The proportional hazards assumption was satisfied (*P* = .1226; Supplementary Figure S1).

**Table 2:** Association between time to first cigarette and incident heart failure

Characteristics	Model 1		*P*-value	Model 2		*P*-value	Model 3		*P*-value
	HR	95% CI		HR	95% CI		HR	95% CI	
Non-smoker	—	—	—	—	—	—	—	—	—
Time from waking to first cigarette (min)									
>120	1.18	0.97–1.44	.104	1.51	1.21–1.87	<.001	1.48	1.06–2.07	<.001
61–120	1.86	1.55–2.22	<.001	1.93	1.59–2.33	<.001	1.86	1.34–2.56	<.001
30–60	2.32	2.10–2.56	<.001	2.07	1.85–2.30	<.001	1.96	1.45–2.62	<.001
5–15	2.54	2.34–2.77	<.001	2.27	2.06–2.50	<.001	2.11	1.56–2.85	<.001
<5	2.79	2.45–3.19	<.001	2.46	2.12–2.84	<.001	2.22	1.58–3.10	<.001
*P* for trend			<.001			<.001			<.001

HR, hazard ratio; CI, confidence interval.

Model 1: unadjusted.

Model 2: adjusted for age, sex, Townsend deprivation index, race, education, BMI, eGFR, WHR, alcohol status, SBP, DBP, hypertension, diabetes, WBC; NEU; LYM; CRP; TG; HbA1c; Glu; LDL-C; healthy sleep status, history of heart attack, angina, history of stroke, insulin use, antihypertensive drugs, and lipid-lowering drugs.

Model 3: Model 2 + daily cigarette smoked and smoking duration.

### Stratified analysis

Age modified the TTFC-HF association (*P*-interaction <.001), with stronger associations in participants <60 years (HR 1.98 [1.56–2.51] for <15-min group) vs ≥60 years (HR 1.55 [1.25–1.91], both compared with non-smokers) (*Table 3*). BMI also modified the TTFC-HF relationship (*P*-interaction = .0013), with normal-weight individuals exhibiting the steepest gradient (HR 1.98 [1.52–2.58] for TTFC <15 min in BMI <25 kg/m^2^ subgroup). No significant interactions were observed for sex, ethnicity, education level, or alcohol consumption status.

**Table 3:** Association of time from waking to first cigarette with the risk of incident heart failure stratified by potential risk factors via model 3

Subgroup	Non-smoker	Time from waking to first cigarette (min)			*P*-trend	*P*-interaction
		>60	30–60	<15		
Age, years						<0.001
<60	1.00 (reference)	1.12 (0.84–1.48)	1.78 (1.42–2.24)	1.98 (1.56–2.51)	0.0000	
≥60	1.00 (reference)	1.68 (1.38–2.03)	1.63 (1.35–1.97)	1.55 (1.25–1.91)	0.0000	
Sex						0.0843
Male	1.00 (reference)	1.14 (0.87–1.48)	1.40 (1.10–1.77)	1.34 (1.04–1.73)	0.0140	
Female	1.00 (reference)	1.58 (1.30–1.92)	1.79 (1.48–2.15)	1.83 (1.49–2.25)	0.0000	
Race						0.8957
White ethnicity	1.00 (reference)	1.41 (1.19–1.66)	1.68 (1.44–1.95)	1.69 (1.43–2.00)	0.0000	
Other ethnicity	1.00 (reference)	1.35 (0.84–2.17)	1.31 (0.8–2.14)	1.18 (0.69–2.01)	0.5059	
Education						0.6287
College university degree	1.00 (reference)	1.35 (1.14–1.61)	1.53 (1.31–1.79)	1.57 (1.33–1.85)	0.0000	
Other level of education	1.00 (reference)	1.46 (1.01–2.11)	1.78 (1.19–2.65)	1.37 (0.82–2.28)	0.0827	
BMI						0.0013
<25	1.00 (reference)	1.34 (0.99–1.83)	1.96 (1.52–2.53)	1.98 (1.52–2.58)	0.0000	
[25, 30)	1.00 (reference)	1.69 (1.33–2.14)	1.66 (1.30–2.12)	1.79 (1.37–2.34)	0.0000	
≥30	1.00 (reference)	1.40 (1.05–1.86)	1.71 (1.32–2.21)	1.71 (1.29–2.26)	0.0001	
Drinking status						0.0512
Never	1.00 (reference)	2.78 (1.32–5.85)	1.67 (0.80–3.51)	1.50(0.70–3.21)	0.3011	
Previous	1.00 (reference)	1.38 (0.72–2.67)	1.41 (0.82–2.44)	1.52 (0.89–2.58)	0.1233	
Current	1.00 (reference)	1.41 (1.19–1.66)	1.67 (1.43–1.95)	1.67 (1.41–1.97)	0.0000	

Estimates are from Cox proportional hazard regression models. Model 3: adjusted for age, sex, Townsend deprivation index, race, education, BMI, eGFR, WHR, alcohol status, SBP, DBP, hypertension, diabetes, WBC; NEU; LYM; CRP; TG; HbA1c; Glu; LDL-C; healthy sleep status, history of heart attack, angina, history of stroke, insulin use, antihypertensive drugs, lipid-lowering drugs, daily cigarette smoked, and smoking duration.

### Joint association of smoking timing, daily cigarette amount, and smoking duration with risk of heart failure

**Figure 3:**
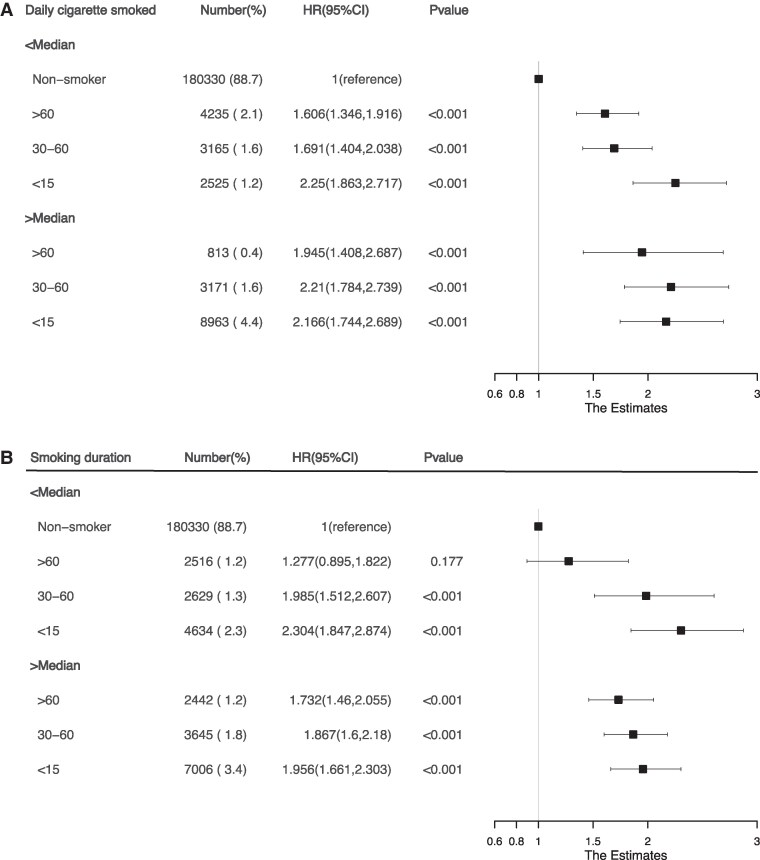
Joint association of smoking timing with (*A*) daily cigarette smoked and (*B*) smoking duration on heart failure risk. Estimates were derived from fully adjusted Cox models (Model 3), controlling for demographic characteristics, clinical risk factors, medication use, and comorbidities. The reference group (HR = 1.0) comprised never-smokers in both panels. The dashed vertical line indicates the reference value

**Figure 4:**
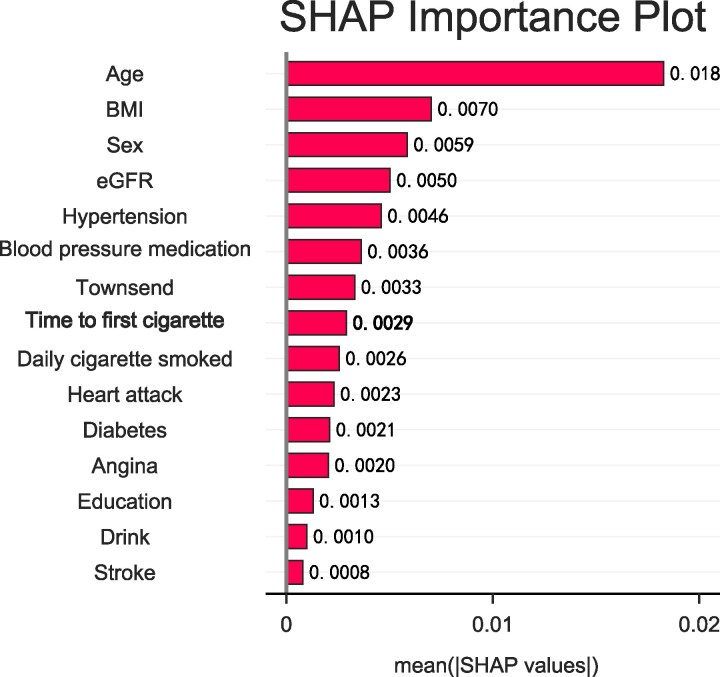
Feature importance ranking from XGBoost machine learning model predicting incident heart failure. Values represent SHAP (SHapley Additive exPlanations) importance scores, with time to first cigarette and daily cigarette consumption emerging as equally strong predictors among the top 15 features

Combined analyses revealed significantly increased risk of incident HF associated with shorter smoking timing after waking, higher daily cigarette consumption, and longer smoking duration across all strata (*Figure 3*). Compared with non-smokers, participants smoking ≤median cigarettes/day, HRs increased from 1.61 (1.35–1.92) to 2.25 (1.86–2.72) across TTFC categories; and those with >median cigarettes/day, corresponding HRs ranged from 1.95 (1.41–2.69) to 2.17 (1.74–2.69). Similar results were observed in the joint analysis of TTFC and smoking duration. A consistent dose-response gradient was evident, showing progressively higher risks with decreasing TTFC. In the feature importance ranking of the XGBoost model, TTFC and daily cigarettes amount had a comparable impact on the risk of incident HF, and both ranked among the top 15 key features (*Figure 4*).

### Sensitivity analyses

Several sensitivity analyses further supported the result robustness in our study. After excluding incident HF cases within two years of follow-up, we confirmed persistent dose-response associations between TTFC and increased HF risk across all models (Supplementary Table S1).Similar dose-response relationships between TTFC and HF risk were also observed after excluding participants with a history of prior heart attack, angina, or prior stroke (Supplementary Tables S2 and S3).

Furthermore, by constructing a competing risk model, with the occurrence of HF regarded as the primary event of interest, all-cause mortality and non-cardiovascular mortality as the competing events, the relationship between TTFC and HF was further investigated. The results showed that TTFC was significantly associated with HF (statistic = 804.3070, df = 5, *P* < .001) and all-cause mortality (statistic = 3425.4835, df = 5, *P* < .001) and showed similar associations when non-cardiovascular mortality was evaluated as a competing event. (Supplementary Tables S4 and S5) The cumulative incidence function plot of the competing risk model further illustrated that shorter TTFC correlated with both higher cumulative incidences of HF and all-cause mortality, confirming the robust association (Supplementary Figure S2).

Results remained consistent when pack-years were used instead of daily cigarette consumption and smoking duration (Supplementary Table S6).

## Discussion

In this large prospective cohort study of UK Biobank participants, we found a dose-dependent association between a shorter TTFC and an increased incidence of HF. Individuals smoking within 5 min of waking exhibited a 2.23-fold higher HF risk compared with never-smokers, even after adjusting for daily cigarette consumption and smoking duration. Notably, this association was particularly strong among younger participants. Moreover, our machine learning analysis positioned TTFC equally with daily cigarette consumption among top predictors. Our findings extend prior research linking TTFC with atrial fibrillation and other cardiovascular outcomes by specifically establishing its predictive value for HF incidence.

Our findings align with but extend previous epidemiological evidence. While the relationship between smoking quantity and HF risk is well-established, our study is among the first to demonstrate that smoking timing independently predicts HF incidence. This complements recent work linking TTFC to atrial fibrillation risk^5^ and aligns with population studies associating shorter TTFC with adverse metabolic profiles^10^ and hypertension^11^—both recognized risk factors for congestive heart failure.^12–14^

Smoking is a recognized independent HF risk factor beyond traditional risk factors,^17–21^ contributing primarily via an increased risk of coronary heart disease.^22^ A Mendelian randomization study confirmed a causal role of smoking in increasing HF risk,^23^ and prior observational studies link greater smoking intensity and cumulative burden to higher HF incidence.^24^ While these studies highlight the broader impact of smoking on HF, the specific relationship between the time of smoking initiation after waking and HF risk has not been clearly defined. Our study addresses this gap by demonstrating an association between TTFC and HF risk.

Three plausible mechanisms may explain our findings (Supplementary Figure S3). First, a shorter TTFC reflects greater nicotine dependence, leading to more intensive smoking behaviours and higher systemic exposure to harmful compounds such as carbon monoxide and reactive oxygen species.^25–30^ These exposures can induce oxidative stress, inflammation, endothelial dysfunction, and renal impairment—key processes involved in HF pathogenesis.^4,30–35^ Second, the rapid nicotine surge upon waking may exacerbate morning cardiovascular stress via acute sympathetic activation and blood pressure elevation, both well-established contributors to HF. Third, circadian disruption may also play a role. Circadian rhythms regulate fundamental physiological and behavioural processes, and their disruption has been linked to metabolic and cardiovascular disorders. Such diurnal variations in blood pressure, heart rate, and vascular function are essential for cardiovascular homeostasis.^36–39^ Early morning smoking may interfere with normal diurnal variations in cardiovascular function regulated by core clock genes (e.g. BMAL1), potentially leading to myocardial metabolic dysregulation and impaired cardiac function over time.^40,41^

Subgroup analyses revealed effect modifications. The TTFC-HF risk association was stronger in participants aged <60 years (e.g. HR = 1.98, <15 min group) than in those ≥60 years (e.g. HR = 1.55, <15 min group), implying TTFC may serve as a relatively independent, potent risk factor earlier in life whereas cumulative vascular insults and multifactorial risks may attenuate its specific contribution in older populations. The effect of TTFC was modified by BMI (*P*-interaction = .0013), with the association most pronounced in normal-weight individuals (BMI < 25) and attenuated but still significant effects among those with higher BMI. Similarly, current drinkers manifested an increase in HF risk with shorter TTFC (*P*-trend <.0001), while no significant trend was observed among never or former drinkers (*P*-trend >.05). These subgroup findings should be interpreted cautiously, as they were exploratory in nature and may be influenced by multiple testing. Replication in independent cohorts is needed to confirm these potential effect modifications.

From a prevention perspective, our findings suggest that assessing TTFC could enhance HF risk prediction, particularly among younger smokers. Incorporating simple questions about morning smoking patterns into clinical encounters may help identify high-risk individuals who could benefit from targeted cessation interventions. Our results also raise the intriguing possibility that behavioural strategies focused specifically on delaying the first cigarette might offer cardiovascular benefits independent of complete cessation—a hypothesis requiring interventional testing. For public health, these findings reinforce the importance of considering temporal patterns of health behaviours in cardiovascular risk assessment.

The study's major strengths include its prospective design, large sample size, comprehensive phenotyping, and rigorous adjustment for potential confounders. Furthermore, the consistent gradient observed across all sensitivity analyses reinforces the robustness of this association and suggests TTFC may represent a clinically useful behavioural biomarker for HF risk stratification.

However, several limitations merit consideration. First, the UK Biobank's volunteer-based recruitment may limit generalizability, though the diversity of smoking behaviours captured mitigates this concern. Second, these categories of TTFC omit the 15–30 min interval (due to an oversight in the design of the initial data collection), however, to ensure analytical consistency, UKB maintained the same response options in subsequent data collections. Third, while we adjusted for numerous confounders, residual confounding remains possible in observational studies. Fourth, the single baseline assessment of smoking behaviour cannot account for changes over time. Additionally, TTFC and smoking characteristics were self-reported, which may introduce recall bias or misclassification. Finally, the predominantly white European population necessitates validation in more diverse cohorts.

Several important questions emerge from these findings. First, Mendelian randomization studies could help establish causality by leveraging genetic variants associated with TTFC. Second, intervention studies should test whether TTFC delay reduces cardiovascular risk markers or HF incidence. Third, research should explore potential gene-environment interactions underlying the observed age-specific effects. Finally, investigation of TTFC's relationship with HF subtypes (HFrEF vs HFpEF) could provide mechanistic insights. Additionally, future studies should further elucidate how TTFC influences myocardial remodelling through sympathetic activation, oxidative stress, and circadian disruption, and consider translational strategies to incorporate TTFC screening into primary care–based smoking cessation programmes.

## Conclusions

This study provides compelling evidence that shorter time to first cigarette is independently associated with a higher risk of incident HF in a dose-dependent manner, with particular relevance for younger adults. By establishing TTFC as a novel behavioural risk factor, these findings suggest that TTFC may serve as a simple behavioural indicator for HF risk assessment, particularly among younger adults. Future research should focus on elucidating underlying mechanisms and testing whether modifying morning smoking patterns can reduce HF risk.

## Supplementary Material

xvag049_Supplementary_Data

## Declarations

### Data Availability

This study used resources from the UK Biobank (approved project number 356 978) and more details on the access process can be found on the UK Biobank website: http://www.ukbiobank.ac.uk/registerapply.
