# Supplementary material for: Time to first cigarette after waking and incident heart failure: a dose-response analysis from the UK biobank
Source: ESC Heart Fail. 2026 Feb 18;13(1):xvag049. doi: 10.1093/eschf/xvag049 (PMC13108290; doi:10.1093/eschf/xvag049)
Supplement: xvag049_Supplementary_Data [file xvag049_supplementary_data.zip › Supplement table(smoking time).docx]

**Supplementary Figure 1.** **Proportional hazards assumption test based on Schoenfeld residuals.**

**
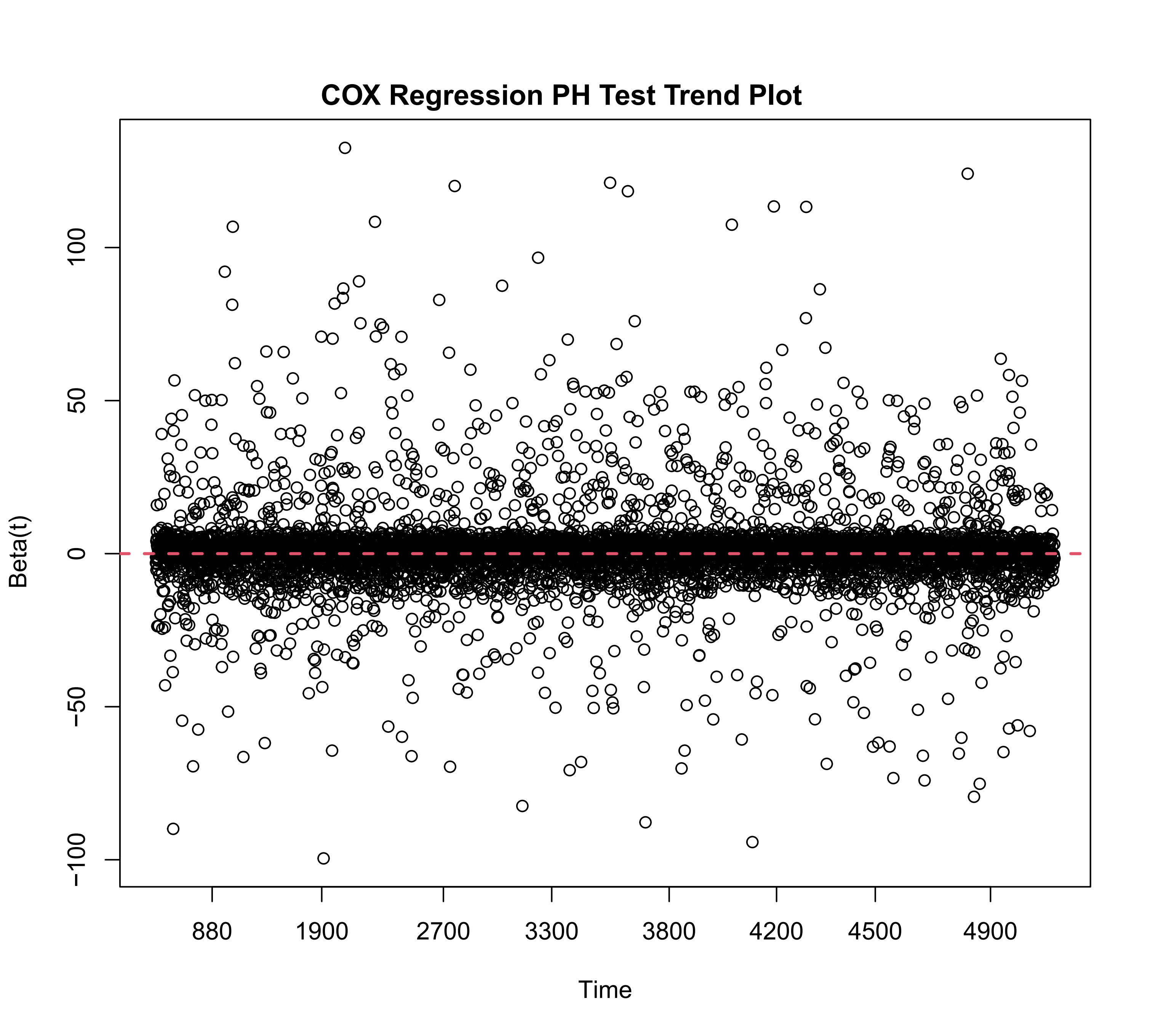
**

**Supplementary Figure 2. Cumulative incidence function (CIF) plots from the competing risk model: (A) TTFC–stratified heart failure and all-cause mortality; and (B) TTFC–stratified heart failure and non-cardiovascular mortality in sensitivity analyses.**

**
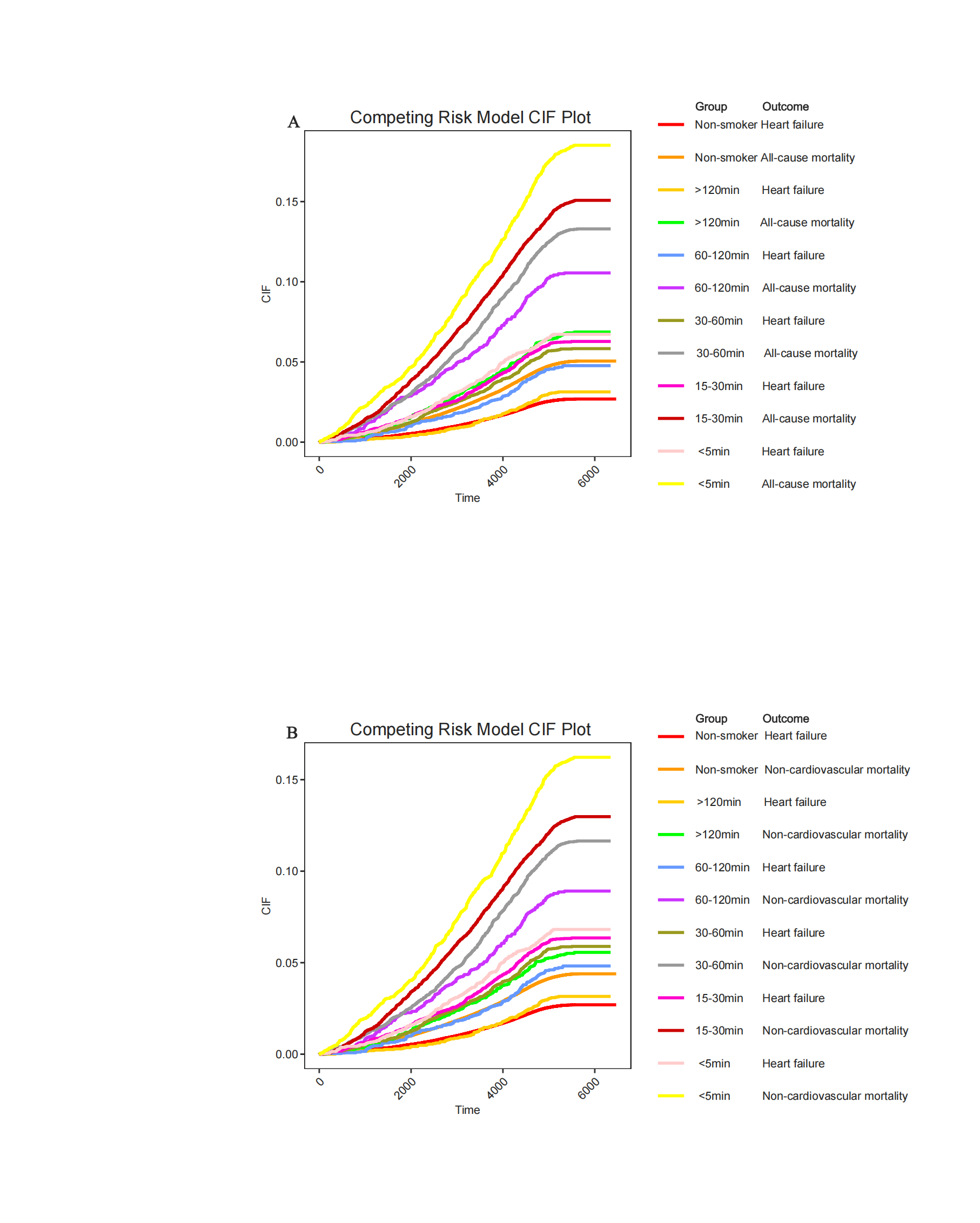
**

| **Supplement Table 1.Sensitivity analysis excluding participants who developed heart failure within 2 years of follow-up.** | | | | | | | | | |
| --- | --- | --- | --- | --- | --- | --- | --- | --- | --- |
| Characteristics | Model 1 | |  | Model 2 | |  | Model 3 | |  |
|  | HR | 95% CI | P-value | HR | 95% CI | P-value | HR | 95% CI | P-value |
| Non-smoker | — | — | — | — | — | — | — | — | — |
| **Time from waking to first cigarette (min)** | | | | | | | | | |
| >120 | 1.18 | 0.95,1.44 | 0.118 | 1.49 | 1.19,1.87 | <0.001 | 1.45 | 1.15,1.83 | <0.001 |
| 61-120 | 1.91 | 1.60,2.30 | <0.001 | 2.02 | 1.67,2.44 | <0.001 | 1.93 | 1.57,2.37 | <0.001 |
| 30-60 | 2.31 | 2.08,2.56 | <0.001 | 2.08 | 1.86,2.33 | <0.001 | 1.96 | 1.68,2.29 | <0.001 |
| 15-30 | 2.51 | 2.29,2.74 | <0.001 | 2.26 | 2.04,2.51 | <0.001 | 2.10 | 1.77,2.48 | <0.001 |
| <5 | 2.76 | 2.41,3.16 | <0.001 | 2.46 | 2.12,2.86 | <0.001 | 2.23 | 1.77,2.80 | <0.001 |
| P for trend |  |  | <0.001 |  |  | <0.001 |  |  | <0.001 |
| HR=Hazard Ratio, CI=Confidence Interval  Model 1:unadjusted;  Model 2: adjusted for Age, Sex, Townsend Deprivation index, Race, Education, BMI, eGFR, WHR, Alcohol status, SBP, DBP, Hypertension, Diabetes,WBC; NEU; LYM;CRP;TG;HbA1c; Glu; LDL-C; Healthy sleep status, History of heart attack, Angina, History of stroke, Insulin use, Antihypertensive drugs, Lipid-lowering drugs.  Model 3: Model 2 + daily cigarette smoked + Smoking duration. | | | | | | | | | |

**Supplementary Figure 3.Proposed pathophysiological pathways linking shorter time-to-first-cigarette (TTFC) with increased risk of heart failure.

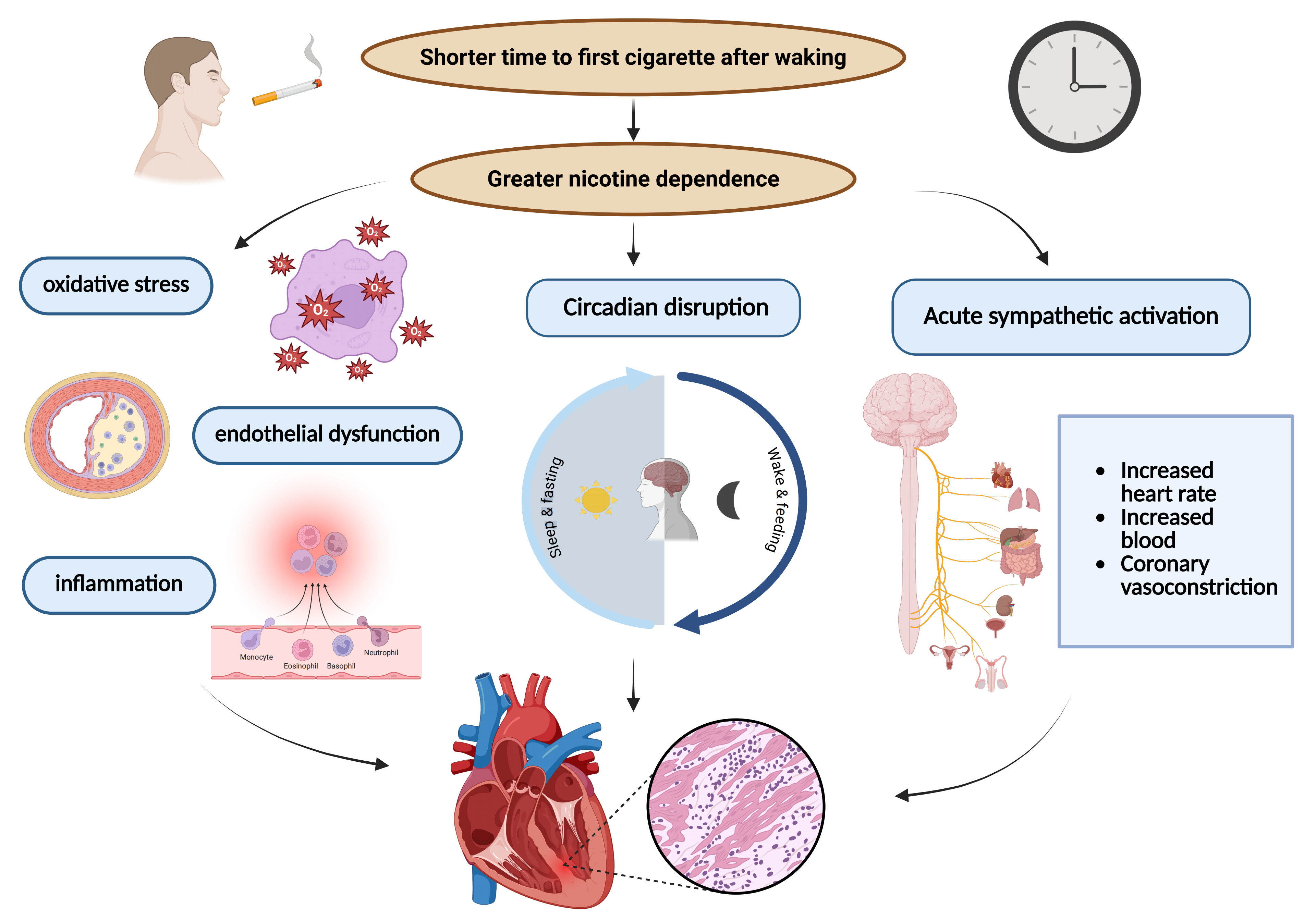
**

| **Supplement Table 2.Sensitivity analysis excluding participants with baseline myocardial infarction or angina.** | | | | | | | | | |
| --- | --- | --- | --- | --- | --- | --- | --- | --- | --- |
| Characteristics | Model 1 | |  | Model 2 | |  | Model 3 | |  |
|  | HR | 95% CI | P-value | HR | 95% CI | P-value | HR | 95% CI | P-value |
| Non-smoker | — | — | — | — | — | — | — | — | — |
| **Time from waking to first cigarette (min)** | | | | | | | | | |
| >120 | 1.22 | 0.99,1.51 | 0.065 | 1.59 | 1.27,2.00 | <0.001 | 1.52 | 1.19,1.92 | <0.001 |
| 61-120 | 1.72 | 1.40,2.09 | <0.001 | 1.86 | 1.51,2.31 | <0.001 | 1.72 | 1.37,2.17 | <0.001 |
| 30-60 | 2.29 | 2.06,2.55 | <0.001 | 2.15 | 1.90,2.43 | <0.001 | 1.94 | 1.65,2.29 | <0.001 |
| 15-30 | 2.42 | 2.19,2.66 | <0.001 | 2.36 | 2.11,2.63 | <0.001 | 2.07 | 1.73,2.48 | <0.001 |
| <5 | 2.65 | 2.29,3.07 | <0.001 | 2.64 | 2.25,3.11 | <0.001 | 2.23 | 1.74,2.86 | <0.001 |
| P for trend |  |  | <0.001 |  |  | <0.001 |  |  | <0.001 |
| HR=Hazard Ratio, CI=Confidence Interval  Model 1: unadjusted;  Model 2: adjusted for Age, Sex, Townsend Deprivation index, Race, Education, BMI, eGFR, WHR, Alcohol status, SBP, DBP, Hypertension, Diabetes,WBC; NEU; LYM;CRP;TG;HbA1c; Glu; LDL-C; Healthy sleep status, History of heart attack, Angina, History of stroke, Insulin use, Antihypertensive drugs, Lipid-lowering drugs.  Model 3: Model 2 + daily cigarette smoked + Smoking duration. | | | | | | | | | |

| **Supplement table 3. Sensitivity analysis excluding participants with baseline stroke** | | | | | | | | | |
| --- | --- | --- | --- | --- | --- | --- | --- | --- | --- |
| Characteristics | Model 1 | |  | Model 2 | |  | Model 3 | |  |
|  | HR | 95% CI | P-value | HR | 95% CI | P-value | HR | 95% CI | P-value |
| Non-smoker | — | — | — | — | — | — | — | — | — |
| **Time from waking to first cigarette (min)** | | | | | | | | | |
| >120 | 1.14 | 0.93,1.41 | 0.118 | 1.48 | 1.18,1.86 | <0.001 | 1.41 | 1.11,1.77 | <0.001 |
| 61-120 | 1.88 | 1.57,2.26 | <0.001 | 2.03 | 1.67,2.46 | <0.001 | 1.87 | 1.52,2.29 | <0.001 |
| 30-60 | 2.29 | 2.06,2.54 | <0.001 | 2.06 | 1.84,2.31 | <0.001 | 1.85 | 1.59,2.16 | <0.001 |
| 15-30 | 2.53 | 2.32,2.76 | <0.001 | 2.28 | 2.06,2.53 | <0.001 | 1.99 | 1.69,2.35 | <0.001 |
| <5 | 2.76 | 2.41,3.16 | <0.001 | 2.58 | 2.22,2.99 | <0.001 | 2.16 | 1.72,2.71 | <0.001 |
| P for trend |  |  | <0.001 |  |  | <0.001 |  |  | <0.001 |
| HR=Hazard Ratio, CI=Confidence Interval  Model 1:unadjusted;  Model 2: adjusted for Age, Sex, Townsend Deprivation index, Race, Education, BMI, eGFR, WHR, Alcohol status, SBP, DBP, Hypertension, Diabetes,WBC; NEU; LYM;CRP;TG;HbA1c; Glu; LDL-C; Healthy sleep status, History of heart attack, Angina, History of stroke, Insulin use, Antihypertensive drugs, Lipid-lowering drugs.  Model 3: Model 2 + daily cigarette smoked + Smoking duration. | | | | | | | | | |

| **Supplement Table 4. Fine-Gray competing risk model for the association between TTFC and HF, with all-cause mortality as a competing event.** | | | |
| --- | --- | --- | --- |
| **Outcome** | Statistics value | P-value | df |
| Heart failure | 804.3070 | <0.001 | 5 |
| All - cause mortality | 3425.4835 | <0.001 | 5 |
| TTFC: Time-to-first-cigarette,HF: Heart Failure  HR=Hazard Ratio, CI=Confidence Interval  Statistics represent likelihood ratio tests for associations between TTFC categories and outcomes. | | | |

| **Supplement Table 5. Fine-Gray competing risk model for the association between TTFC and HF, with Non-cardiovascular mortality as a competing event.** | | | |
| --- | --- | --- | --- |
| **Outcome** | Statistics value | P-value | df |
| Heart failure | 822.2134 | <0.001 | 5 |
| Non-cardiovascular mortality | 2916.3107 | <0.001 | 5 |
| TTFC: Time-to-first-cigarette,HF: Heart Failure  HR=Hazard Ratio, CI=Confidence Interval  Statistics represent likelihood ratio tests for associations between TTFC categories and outcomes. | | | |

| **Supplementary Table 6. Sensitivity analysis of TTFC and incident heart failure using pack-years in place of daily cigarette consumption and smoking duration** | | | | | | | | | |
| --- | --- | --- | --- | --- | --- | --- | --- | --- | --- |
| Characteristics | Model 1 | |  | Model 2 | |  | Model 3 | |  |
|  | HR | 95% CI | P-value | HR | 95% CI | P-value | HR | 95% CI | P-value |
| Non-smoker | — | — | — | — | — | — | — | — | — |
| **Time from waking to first cigarette (min)** | | | | | | | | | |
| >120 | 1.18 | 0.97-1.44 | 0.104 | 1.51 | 1.21-1.87 | <0.001 | 1.49 | 1.19-1.86 | <0.001 |
| 61-120 | 1.86 | 1.55-2.22 | <0.001 | 1.93 | 1.59-2.33 | <0.001 | 1.89 | 1.54-2.31 | <0.001 |
| 30-60 | 2.32 | 2.10-2.56 | <0.001 | 2.07 | 1.85-2.30 | <0.001 | 2.01 | 1.74-2.32 | <0.001 |
| 15-30 | 2.54 | 2.34-2.77 | <0.001 | 2.27 | 2.06-2.50 | <0.001 | 2.19 | 1.88-2.55 | <0.001 |
| <5 | 2.79 | 2.45-3.19 | <0.001 | 2.46 | 2.12-2.84 | <0.001 | 2.34 | 1.90-2.89 | <0.001 |
| P for trend |  |  | <0.001 |  |  | <0.001 |  |  | <0.001 |
| HR=Hazard Ratio, CI=Confidence Interval  Model 1:unadjusted;  Model 2: adjusted for Age, Sex, Townsend Deprivation index, Race, Education, BMI, eGFR, WHR, Alcohol status, SBP, DBP, Hypertension, Diabetes,WBC; NEU; LYM;CRP;TG;HbA1c; Glu; LDL-C; Healthy sleep status, History of heart attack, Angina, History of stroke, Insulin use, Antihypertensive drugs, Lipid-lowering drugs.  Model 3: Model 2 + pack-years | | | | | | | | | |
