# Supplementary material for: Time to first cigarette after waking and incident heart failure: a dose-response analysis from the UK biobank
Source: ESC Heart Fail. 2026 Feb 18;13(1):xvag049. doi: 10.1093/eschf/xvag049 (PMC13108290; doi:10.1093/eschf/xvag049)
Supplement: xvag049_Supplementary_Data [file xvag049_supplementary_data.zip › SupplementaryFig3.pdf.pdf]

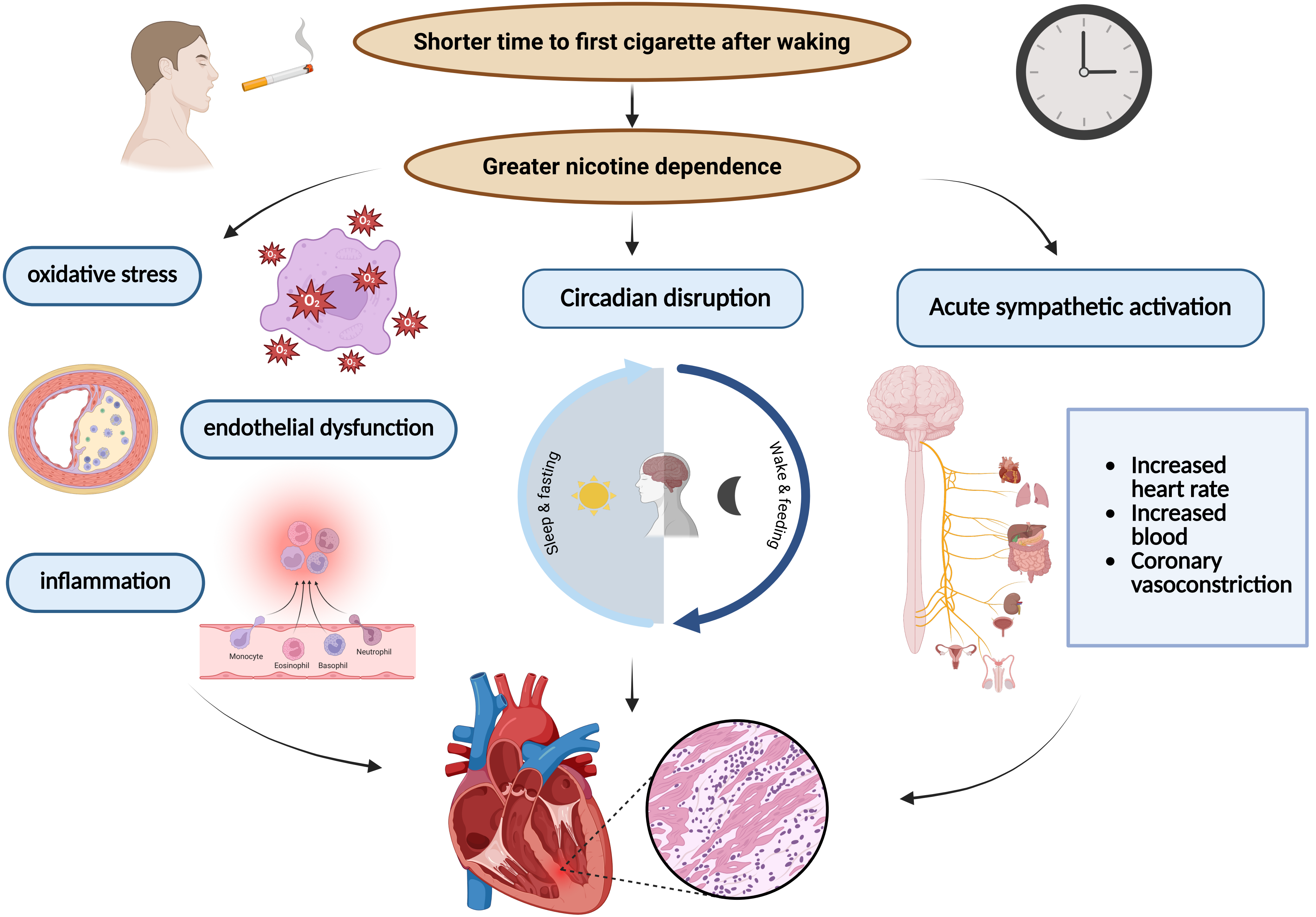

Shorter time to first cigarette after waking
Greater nicotine dependence
oxidative stress
Circadian disruption
Acute sympathetic activation
endothelial dysfunction
Sleep & fasting
Wake & feeding
inflammation
Monocyte
Eosinophil
Basophil
Neutrophil
Increased heart rate
Increased blood
Coronary vasoconstriction
