# Supplementary figures and images for: Time to first cigarette after waking and incident heart failure: a dose-response analysis from the UK biobank
Source: ESC Heart Fail. 2026 Feb 18;13(1):xvag049. doi: 10.1093/eschf/xvag049 (PMC13108290; doi:10.1093/eschf/xvag049)

COX Regression PH Test Trend Plot

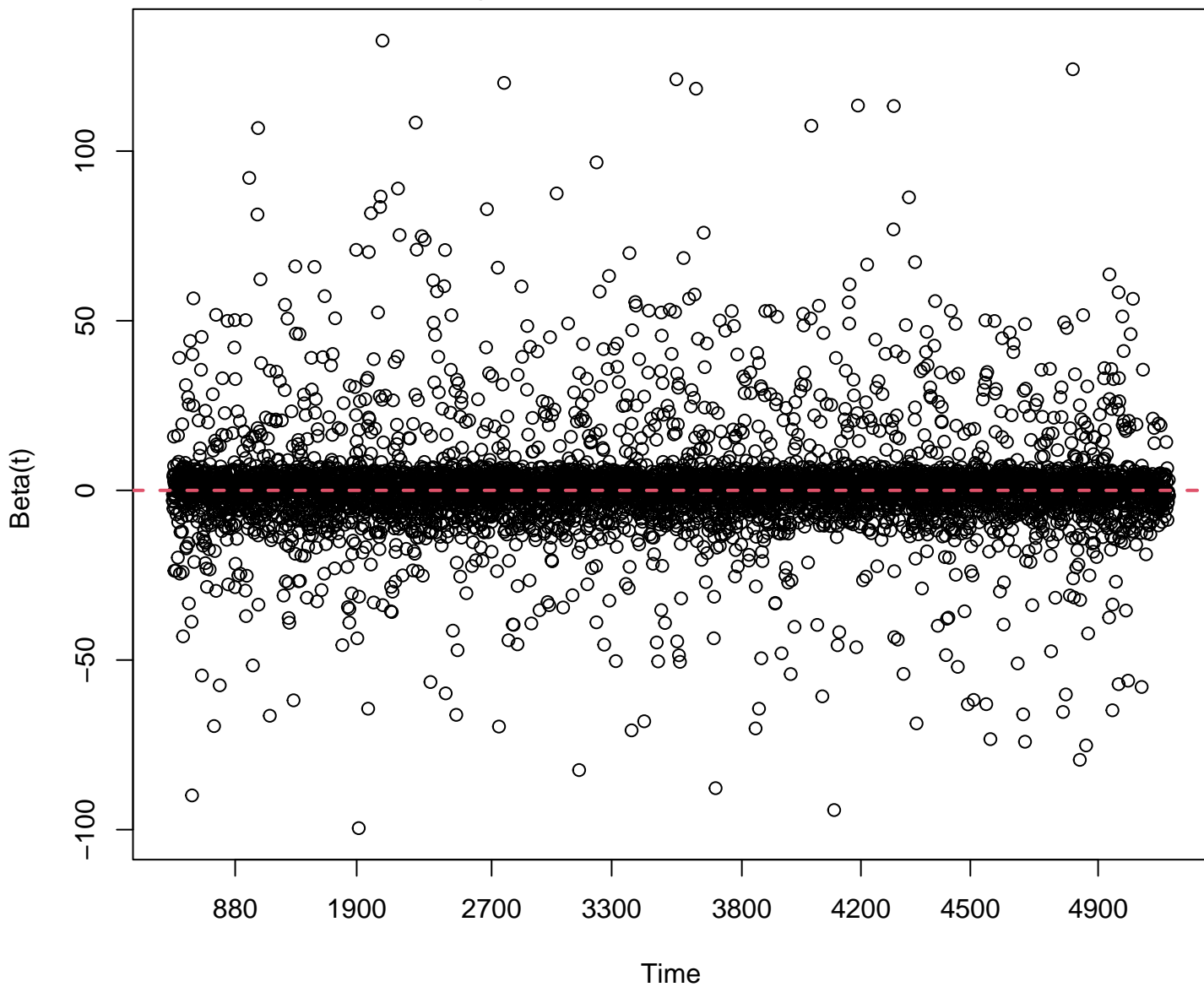

Supplement: xvag049_Supplementary_Data [file xvag049_supplementary_data.zip › SupplementaryFig1.pdf.pdf]

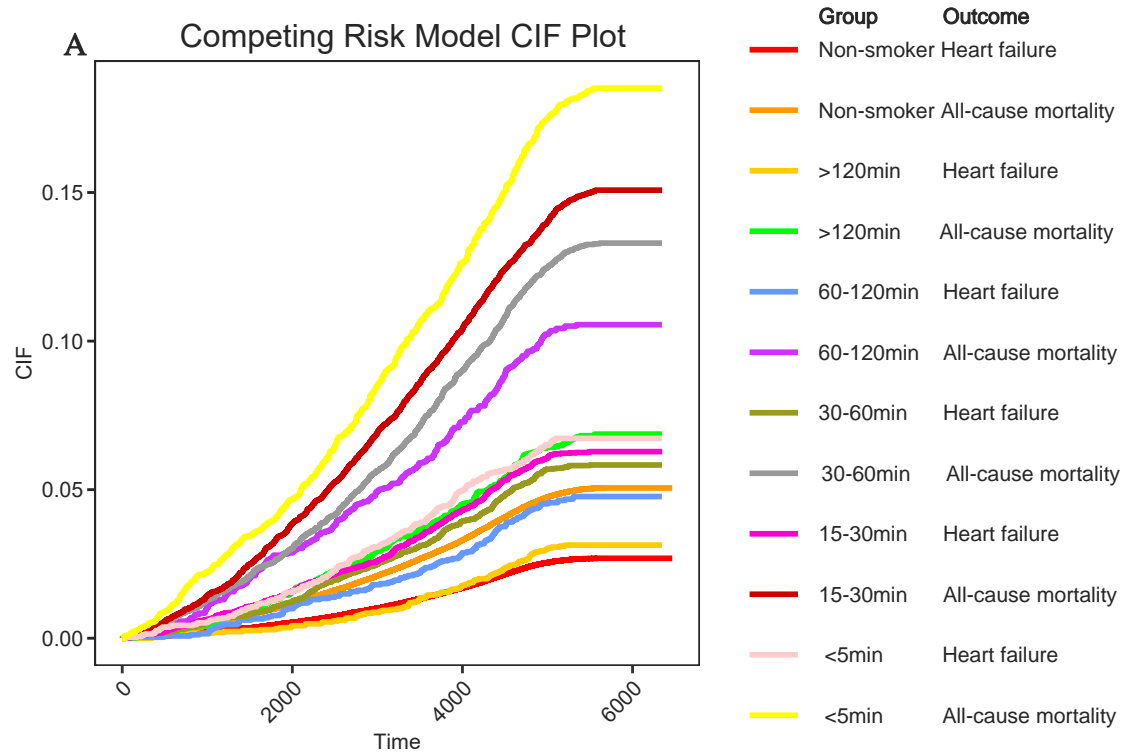

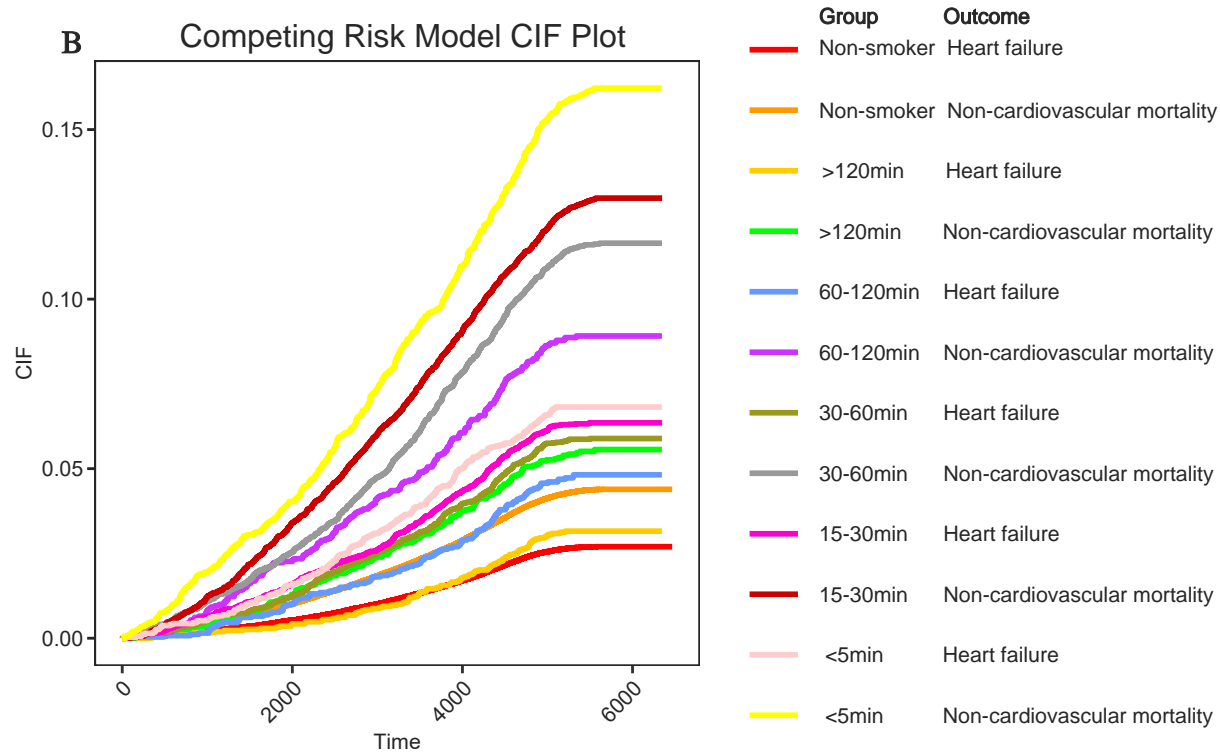

Supplement: xvag049_Supplementary_Data [file xvag049_supplementary_data.zip › SupplementaryFig2.pdf.pdf]
